# Dr. Henry Collidge Frost

**Published:** 1916-09

**Authors:** 


**Dr. Henry Collidge Frost,** Cleveland Homoeopathic 1874, of Buffalo, died in Montreal, July 25.

